# Photodynamic Pattern Memory Surfaces with Responsive Wrinkled and Fluorescent Patterns

**DOI:** 10.1002/advs.202002372

**Published:** 2020-10-14

**Authors:** Shuai Chen, Tianjiao Ma, Jing Bai, Xiaodong Ma, Jie Yin, Xuesong Jiang

**Affiliations:** ^1^ School of Chemistry & Chemical Engineering Frontiers Science Center for Transformative Molecules State Key Laboratory for Metal Matrix Composite Materials Shanghai Jiao Tong University Shanghai 200240 P. R. China

**Keywords:** dynamic wrinkles, near‐infrared regulation, pattern memory surfaces, reversible fluorescence, supramolecular network

## Abstract

Reversible pattern systems, namely pattern memory surfaces, possessing tunable morphology play an important role in the development of smart materials; however, the construction of these surfaces is still extensively challenging because of complicated methodologies or chemical reactions. Herein, a functionalized basement is strategically integrated with a multi‐responsive supramolecular network based on hydrogen bonding between aggregation‐induced emission luminogens (AIEgens) and copolymers containing amidogen (poly(St‐*co*‐Dm) to establish a bilayer system for near‐infrared (NIR)‐driven memory dual‐pattern, where both the fluorescence emission and wrinkled structures can be concurrently regulated by a noninvasive NIR input. The motion of the AIEgens and photo‐to‐thermal expansion of the modified base allow temporal erasing of the fluorescent wrinkling patterns. Meanwhile, when exposed to 365 nm UV radiation, the fluorescent patterns can be independently regulated through photocyclization. The fluorescent wrinkling pattern presented herein is successfully demonstrated to promote the level of information security and capacity. This strategy provides a brand‐new approach for the development of smart memory interfaces.

## Graphical abstract

A broad‐spectrum strategy for fabricating photodynamic memory patterns is presented by integrating aggregation‐induced emission luminogen into copolymer to form supramolecular network. Upon exposure to non‐invasive near‐infrared light, the proposed dual‐pattern system can afford temporal erasure toward fluorescence and wrinkled structures, thereby promoting information security and capacity.

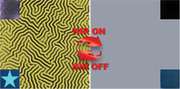

Inspired by the profound comprehension of natural and living organisms, the development of patterned surfaces has become a fundamentally important element in both basic subjects and practical applications.^[^ ^1^ ^]^ Particularly, similar to shape‐memory polymers,^[^ ^2^ ^]^ a class of pattern memory surfaces (PMSs) whose morphology can completely return to its original permanent status reversibly upon external stimulation has attracted significant attention in the last few years because the dynamic change in morphology from nano‐ to microscale can provide on‐demand control of surface properties including switchable wetting,^[^ ^3^ ^]^ adhesion control,^[^ ^4^ ^]^ intelligent displays,^[^ ^3^, ^5^ ^]^ and signal delivery.^[^ ^6^ ^]^ The PMSs presented herein can play an important role in the development of next‐generation smart surfaces. In the previously proposed PMSs, aside from the shape and altitude variation in their topological structures, another notable aspect is that the reversible morphology generally focuses on the color/fluorescence or intensity change. Compared to topography memory, reversible fluorescence is slightly easy to realize via intentional molecular chemistry design, which enables a broad range of promising applications of PMSs in data recording, information security, sensors, and so on.^[^ ^7^ ^]^ However, there are still enormous challenges in the development of a robust approach for topography memory patterns possessing dynamic tunability due to the microscopic motion of macromolecular chains and interactions caused by substrates.

Accordingly, as a naturally mechanical phenomenon, wrinkled patterning on film–substrate bilayer systems^[^ ^8^ ^]^ resulting from compressive stress provides an intriguing avenue for overcoming the abovementioned obstacles and has attracted considerable research interest owing to the distinguished optical,^[^ ^9^ ^]^ electrical,^[^ ^10^ ^]^ biological,^[^ ^11^ ^]^ and mechanical^[^ ^9^, ^12^ ^]^ performances of the developed patterns. To date, manipulating the progress of stress relaxation and modulus within the wrinkled topologies based on indirect or direct actuation (e.g., light, temperature, pH, and solvents) has enabled the realization of dynamically tunable smart surfaces.^[^ ^8a^ ^]^ Nevertheless, most of the PMSs reported to date face problems due to single morphology memory and require invasive stimuli because of the sophisticated preparation methodologies and molecular‐level material chemistries.^[^ ^13^ ^]^ Moreover, an ideal photodynamic strategy, especially near‐infrared (NIR)‐induced stimulation capable of noninvasive and remote regulation, which is previously seldom established, is indeed anticipated to enable the temporal alternative tuning of memory appearances and properties.

Herein, we present the facile yet robust fabrication of a dynamically synergistic NIR‐responsive fluorescent image with wrinkled topography for the first time, whose significant real‐time response is favorable to achieve multidirectional PMSs (**Figure** **1**). Accordingly, herein, a supramolecular network comprising hydroxyl‐containing aggregation‐induced‐emission luminogens (AIEgens)^[^ ^14^ ^]^ and an amine‐based copolymer is intentionally spin‐coated on polydimethylsilane‐containing carbon nanotube (CNT‐PDMS) elastomer substrates. In this strategy, the noteworthy stimuli‐responsive hydrogen bonding in the supramolecular network plays the following roles: i) acts as the key point of controlling the cross‐linking density and moduli of the top stiff layer; ii) affects the intramolecular motions of the phenyl rings of AIEgens; iii) helps the patterned surface to be reversible based on the dynamic chemistry; and iv) enables a sensitive response to temperature and acid gases. Additionally, unordered fingerprint‐like wrinkles with various high‐resolution fluorescent drawings were successfully obtained owing to the photocyclization of tetraphenylethene (TPE),^[^ ^6^, ^15^ ^]^ promoting the information capacity and security of patterns. The resulting pattern with wrinkled topography and blue fluorescence can be temporally erased *in situ* by NIR irradiation due to the photo‐to‐thermal conversion of CNTs, allowing the optional alteration of the patterned multiple security information over great distances.

**Figure 1:**
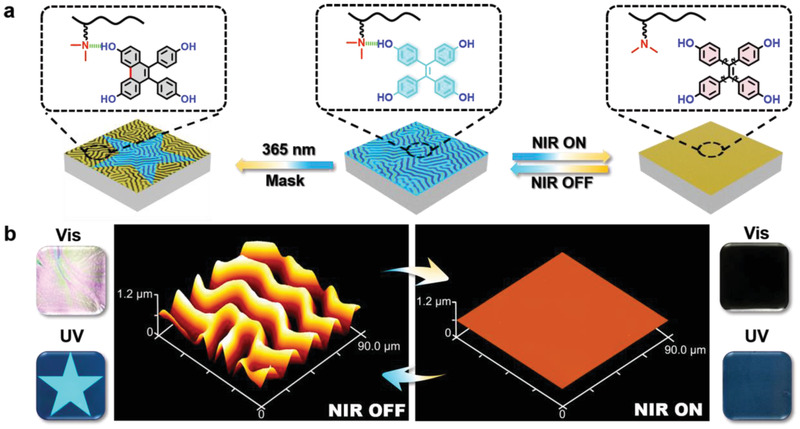
Proposed strategy for the establishment of the photodynamic PMS with tunable properties. a) Schematic illustration exhibiting chemical conversions for the controlled morphology in response to UV or NIR light. b) AFM images and macroscopic images demonstrating the reversibility of the NIR‐driven dual‐pattern. The CNTs content in the elastic base is 0.5 wt‰, and the NIR intensity is ≈1.5 W cm^−2^.

The use of wrinkled surfaces possessing micro‐ and nano‐pattern topologies with spatially periodic or aperiodic structures, which are responsive to noninvasive excitation such as temperature and light, for the formation of novel memory patterns has been a promising strategy. The wrinkling of thin supramolecular networks above elastic bases is a significant alternative to various technologies due to natural generation, versatility, and sensitivity of the patterns.^[^ ^8b^ ^]^ In this respect, adjusting the multi‐responsive chemical bonds for controlling the modulus and cross‐linking density of the supramolecular network is the key point in forming wrinkling patterns.^[^ ^16^ ^]^ To synergistically realize controlled fluorescence intensity, the strategical integration of one kind of unique luminophores, whose emission can be observably transformed by tuning the intermolecular interactions within the supramolecular network, is urgently required. Considering the fact that the emission of TPE derivatives is usually reversible based on the restriction of intramolecular motion (RIM), which might infuse the supramolecular network with responsive fluorescence, the use of TPE derivatives for the facile fabrication of PMSs with reversible wrinkled and fluorescent patterns is a significant strategy. The evolution of the fluorescent wrinkles, whose topology structure was observed via atomic force microscopy (AFM), upon switching off the NIR source and the fluorescent pattern with reversible tunability simultaneously affirmed our supposition on photodynamic memory patterning (Figure 1b).

In our strategy, a typical AIEgen, that is, the TPE derivative TPEOH with four hydroxyl groups, was chosen and directly synthesized according to the McMurry coupling reaction (Scheme S1, Supporting Information).^[^ ^17^ ^]^ The successful preparation of TPEOH was confirmed by ^1^H nuclear magnetic resonance (^1^HNMR) spectroscopy (Figure S1, Supporting Information). Moreover, the random copolymers poly(St‐*co*‐Dm) containing 2‐dimethylaminoethyl acetate in the side chain, which could regulate the obtained macromolecular modulus and simultaneously assemble TPEOH through dynamic hydrogen bonding, were synthesized by free‐radical polymerization according to Scheme S2, Supporting Information. The assignment of the proton peaks in the ^1^H NMR spectrum of poly(St‐*co*‐Dm) [average *M*_n_ = 16.7 kDa; *M*_w_/*M*_n_ = 1.47] demonstrated that the styrene:2‐dimethylaminoethyl acetate molar ratio was ≈1.6:1 (Figure S2 and Table S1, Supporting Information). As predicted, strong blue fluorescence could be obtained by gradually increasing the content of the poor solvent for limiting the molecular motion of TPEOH (Figure S3, Supporting Information). The fluorescence spectra of the poly(St‐*co*‐Dm)@TPEOH supramolecular network also verified the effective association between TPEOH and poly(St‐*co*‐Dm), leading to the RIM of TPEOH and intense fluorescence (Figure S4, Supporting Information). The peak at 3420 cm^−1^ in the Fourier transform infrared spectra assigned to the O‐H stretching vibration weakened and shifted to higher wavenumbers with the increasing temperature; this further confirmed the occurrence and typical temperature sensitivity of the hydrogen bonding between poly(St‐*co*‐Dm) and TPEOH (Figure S5, Supporting Information).^[^ ^16^ ^]^ These results suggested that the intentional introduction of distinctive AIEgens into a supramolecular network might lead to a reversible fluorescent wrinkling bilayer.

To obtain detailed insight into the proposed system and thus utilize its distinct features, topological evaluations and fluorescence spectroscopy of the supramolecular films were conducted to preliminarily investigate our speculation. The mixed toluene/THF (1:1) solution comprising poly(St‐*co*‐Dm)@TPEOH (the molar ratio of tertiary amine to hydroxyl group is 5:1) was spin‐coated onto the CNT‐PDMS (0.5 wt‰) elastomer as a soft upper layer. Upon thermal treatment at 70 °C for 3 min and subsequent cooling to room temperature, an unordered wrinkling pattern spontaneously appeared in the established bilayer system, which was attributed to the release of localized stress between layers (Figure S6, Supporting Information). Notably, the exhibited pattern of random, unpredictable, and distinct wrinkles also possessed both cyan fluorescence and a biomimetic fingerprint‐like character (i.e., ridge ending and bifurcation).^[^ ^9^, ^18^ ^]^ Owing to the lack of TPEOH and supramolecular cross‐linking, the surface of the individual poly(St‐*co*‐Dm)‐coated CNT‐PDMS elastomer exhibited no obvious fluorescence and wrinkling pattern after undergoing the same procedures; this indicated that the TPEOH trigger is effective for the generation of fluorescence and wrinkles.

Additionally, TPE and poly(St‐*co*‐Ba) were synthesized according to the abovementioned methods for comparison. Compared to the poly(St‐*co*‐Dm)@TPEOH complex film, the poly(St‐*co*‐Dm)@TPE bilayer emitted very weak fluorescence upon UV excitation at room temperature because of the absence of hydrogen bonding interactions, which tremendously restrict the intramolecular rotation and vibration of AIEgens (Figure S6c, Supporting Information). Simultaneously, the mechanical properties of the thin coating changed. According to the typical linear buckling theory,^[^ ^8b^ ^]^ the generation of wrinkles in bilayer systems depends on both the modulus and thickness of the hard top layers. The absence of hydroxyl groups altered the cross‐linking density of the thin superstratum such that the poly(St‐*co*‐Dm)@TPE skin could not achieve enough modulus to generate a wrinkled pattern. Furthermore, the poly(St‐*co*‐Ba)@TPEOH film remained smooth after thermal treatment; this further affirmed the certain significance of the cross‐linking density based on chemical hydrogen bonding in the proposed skin layer. Therefore, it is reasonable to believe that tuning the morphologies of fluorescent wrinkles is possible by regulating the dynamic chemistry in bilayer systems.

The poly(St‐*co*‐Dm)@TPEOH supramolecular network with diverse sensitivities used for fabricating the ground‐breaking AIE‐loaded wrinkling surfaces might be applied to realize smart optical and topological memory materials. Subsequently, to investigate the molecule‐driven mechanism of the fluorescent wrinkling surface, laser scanning confocal microscopy (LSCM) was employed to study the evolution of the patterned surface morphology. Note that the hydrogen bond between poly(St‐*co*‐Dm) and TPEOH is sensitive to some external stimuli such as heat and acid gases.^[^ ^19^ ^]^ Hence, the reversible cross‐linking attributed to hydrogen bonding in the supramolecular network might be utilized to establish multifunctional acid‐responsive and thermosensitive wrinkle patterns. Under a volatile hydrochloric acid (HCl) atmosphere, where the partial pressure of HCl is ≈894 ppm, the wrinkled surface gradually recovers to a smooth state (Figure S7, Supporting Information). The LSCM images revealed that the characteristic amplitude (*A*) of the wrinkles reduced from the original value of 907 to 130 nm after acid treatment for 3 min; this suggests that the fabricated wrinkled pattern can be hopefully used for detecting HCl steam. The elimination of wrinkles was ascribed to the destruction of hydrogen bonds by HCl and the consequent damage of the cross‐linking network in the skin, followed by the release of the compressive stress of the bilayer. Additionally, a constant decrease in the fluorescence intensity of the poly(St‐*co*‐Dm)@TPEOH film was observed due to the enhanced intramolecular motions of TPEOH after continuous acid treatment caused by the liberation of TPEOH from poly(St‐*co*‐Dm)@TPEOH; this once again confirmed the previous proposal (Figure S8, Supporting Information). After heating the film to exclude acid, the smooth surface recovered to the wrinkled state due to the dynamic hydrogen bonding interactions.

As the abovementioned thermosensitive hydrogen bond is the key point in arranging the cross‐linking density, modulus, and fluorescence of the supramolecular network, it is promising to utilize it to fabricate multiple memory surfaces. Herein, the fluorescence spectra (Ex = 330 nm) of the 1 wt% TPEOH‐doped copolymer at various temperatures from 30 to 90 °C with an interval of 5 °C were measured (**Figure** **2**a). As the temperature increased, an apparently consistent drop in the fluorescence intensity of the TPEOH‐loaded film was observed possibly because the rigid supramolecular matrices and stable hydrogen bond restrict the intramolecular motions of the phenyl rings of TPEOH, and thus, TPEOH emits efficient fluorescence at low temperatures. Subsequently, the extended space in the supramolecular network released by the movement of rigid polymeric matrices segments and the weakened hydrogen bonding activate the intramolecular motions of TPEOH with a further increase in temperature; this process consumes the energy of the excited state, leading to the decreased fluorescence intensity of the constructed material.^[^ ^20^ ^]^

**Figure 2:**
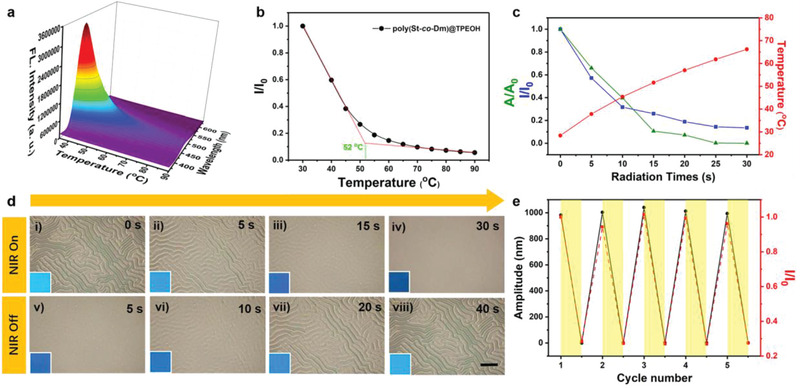
Dynamically tunable dual‐pattern evolution behavior induced by temperature or NIR exposure: a) Fluorescence spectra of the 1 wt% TPEOH‐containing network at a series of temperatures. Excitation: 330 nm. b) Fluorescence intensity plots of the poly(St‐*co*‐Dm)@TPEOH film at 460 nm as a function of increasing temperatures normalized to 30 °C. Excitation: 330 nm. c) Amplitude (green triangle, left vertical axis), fluorescence intensity (at 460 nm, blue square, left vertical axis), and temperature (red circle, right vertical axis) of the bilayer as a function of NIR irradiation for various times. d) Optical images of the reversible erasure/generation behavior of wrinkles under NIR irradiation, and the insets show the corresponding images of the film irradiated by a UV lamp (365 nm). Scale bar: 100 µm. e) NIR‐driven erasure/formation cycles of amplitude with fluorescence. The CNTs content in the elastic base is 0.5 wt‰, and the NIR light intensity is 1.5 W cm^−2^.

Furthermore, the fluorescence emission of TPEOH provides another valuable information on the practical connection of TPEOH with poly(St‐*co*‐Dm). The fluorescence intensity plots at 460 nm against temperature, which were normalized to 30 °C, demonstrated the occurrence of a transition between 50 and 60 °C (Figure 2b). According to the intersection of the two tangent lines, the turning point is confirmed at ≈52 °C, which is close to the glass transition temperature (*T*_g_) of poly(St‐*co*‐Dm) detected by differential scanning calorimetry (Figure S9, Supporting Information). Moreover, the addition of a small quantity of TPEOH did not significantly affect the *T*_g_ of poly(St‐*co*‐Dm). That is, the abovementioned fluorescence strategy may provide deep insights into supramolecular materials in a facile, reliable, and straightforward manner.

Apart from showing thermal‐responsiveness, the morphology of the bilayer can respond to NIR inputs. Herein, to endow the elastomeric substrates with dynamic NIR sensitivity, a typical photo‐to‐thermal conversion material, that is, single‐walled CNTs, was intentionally introduced into the PDMS sheets according to our previous work.^[^ ^5^ ^]^ The photo‐to‐thermal conversion efficiency of the CNT‐PDMS elastomer can be controlled by tuning the contents of CNTs or NIR irradiation intensity. Under NIR irradiation (808 nm, 1.5 W cm^−2^), the temperature of the PDMS elastomer doped with a small amount of CNTs (0.5 wt‰ CNT‐PDMS) rapidly increased and reached above 100 °C within two min (Figure S10, Supporting Information). Contrary to the case of the pure PDMS base without CNTs, whose surface temperature remained constant under NIR irradiation, the incorporation of CNTs markedly promoted the controllability over the established dual‐pattern.

Note that the NIR‐driven pattern surface, which differs from that of conventional unchanging materials, provides a distinctive and effective approach to tune the proposed thermosensitive memory surfaces. For example, the fluorescent wrinkles on the constructed bilayer system can also be temporally erased via NIR irradiation. The wrinkled topography gradually faded with continuous NIR irradiation and completely disappeared within 30 s of irradiation (Figure 2c,d and Movie S1, Supporting Information). The main driving force for this is believed to be the thermal expansion and shape change of the CNT‐PDMS elastomer upon exposure to NIR light, which is attributed to the powerful photo‐to‐thermal conversion capability of CNTs. The thermal expansion arising from NIR exposure gradually reached and exceeded the critical elimination threshold, resulting in the removal of wrinkles. Meanwhile, the fluorescence intensity and characteristic parameter *A* of the dual‐pattern concurrently decreased, which further demonstrated the external long‐distance, robust, and noninvasive adjustability of the patterned surface (Figure 2d). After removing the NIR source and cooling the bilayer system, the smooth surface recovered to the original wrinkled state and exhibited cyan fluorescence, affirming the temporal photodynamic processability of the dual‐pattern. Furthermore, it is inspiring that the reversible disappearance/formation of the fluorescent wrinkles can be achieved for a series of cycles after switching on/off the NIR source, respectively (Figure 2e; Figures S11 and S12, Supporting Information). These results demonstrate an interesting and reliable approach for the fabrication of NIR‐responsive memory surfaces.

Thin films having micro/nanoscale wrinkled structures with regionally patterned fluorescence are fundamentally important for the development of novel man‐made materials used in intelligent sensing systems, smart displays, optical devices, and anti‐counterfeiting.^[^ ^9^, ^21^ ^]^ Considering that the proposed PMSs has multi‐stimuli sensitivity and robust accessibility, its potential applications in light direct writing for improved image capacity were explored. As a classical AIE fluorophore with a structure similar to that of *cis*‐stilbene, TPE and its derivatives were subjected to photocyclization and subsequent oxidation to form the stable fluorophore phenanthrene under UV irradiation (Figure S13, Supporting Information).^[^ ^6^, ^15^ ^]^ As illustrated in the UV–vis spectra, the emergence of a red‐shift of the ultraviolet spectrum attributed to the enhanced conjugation confirmed the transformative structure of TPEOH. With the increasing UV irradiation (365 nm) duration, the fluorescence intensity of the exposed areas decreased in situ, which was significantly different from the case of the unexposed areas (**Figure** **3** and Figure S13c, Supporting Information). After subjecting the CNT‐PDMS elastomer to straightforward UV irradiation through a photomask in air, a 2D fluorescence patterned bilayer system with luminous drawing and clear outline, observed by super‐resolution multiphoton confocal microscopy (STED), could be directly prepared. A series of well‐resolved wrinkled surfaces containing different fluorescent images and disordered topological structures were readily fabricated in a similar manner using 365 nm UV radiation through various shapes of the photomask (Figure 3d). More interestingly, the emission and topological structures of the wrinkled surfaces with a cyan five‐pointed star pattern, whose fluorescence was selectively erased by UV light, could be temporally covered/revealed by switching on/off the NIR source due to molecular rotation and thermal expansion; this suggests that a photo‐driven pattern surface was successfully achieved (Figure S14, Supporting Information).

**Figure 3:**
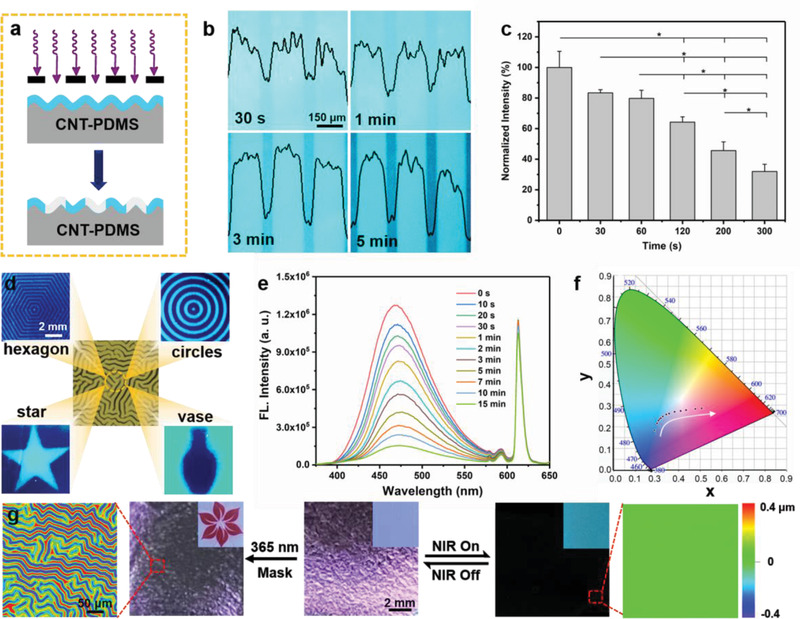
Evolution of the on‐demand fluorescence deployed within the wrinkled surface. a) Rational design of the dual‐pattern exhibiting selective fluorescence under 365 nm UV radiation. b) Time‐dependent fluorescent images with the corresponding intensity profiles and c) fluorescent ratio (*p < 0.05) of the top layer obtained by STED under UV irradiation for different times (365 nm). Results are shown as mean ± SD (*n* = 4). Scale bar: 150 µm. d) Images of the cyan fluorescent pattern obtained upon 365 nm UV exposure through a varied photomask for 6 min. Scale bar: 2 mm. e) Fluorescence emission spectra of the Eu^III^‐functionalized supramolecular network and f) its corresponding fluorescence emission color (CIE color space) after exposure to UV irradiation for a series of increasing times. Excitation: 340 nm. g) Optical and LSCM images of the dual‐pattern system after UV or NIR light (808 nm) treatment. Scale bars: 50 µm and 2 mm. The UV light intensity is 15 mW cm^−2^, and the NIR light intensity is 1.5 W cm^−2^.

We wondered whether the recommended approach of in situ photo drawing could help construct multicolor patterns based on the fading effect of AIEgens. Considering the long lifetime, high luminescence efficiency, and photostability of lanthanide complexes (e.g., Tb^III^ and Eu^III^ chelates),^[^ ^22^ ^]^ who have attracted substantial research attention, the widely utilized Eu^III^ (EuTTA) complex was introduced into the multifunctional PMSs system (Scheme S3, Supporting Information).^[^ ^22b^ ^]^ The cool tone of cyan fluorescence observed from the EuTTA‐functionalized poly(St‐*co*‐Dm)@TPEOH (poly@TPEOH@Eu) film noticeably transformed to pink with the increasing EuTTA content (Figure S15, Supporting Information).

Subsequently, the alternative fading of TPEOH moieties according to their exposure time to 365 nm UV irradiation was confirmed by recording the fluorescence spectra of a thin poly@TPEOH@Eu film (Figure 3e). The fluorescence intensity at 470 nm, attributed to TPEOH, under extended UV exposure reduces over time, indicating the continuous conversion of TPEOH. Compared to the sharply decreased intensity of the TPEOH peak, the emission peak intensity of the Eu^III^ complex at 620 nm remained constant during the 365 nm irradiation. Moreover, the CIE color space coordinates show that an apparent fluorescence change from sky‐blue to aubergine occurs, confirming that tunable fluorescence patterning is feasible (Figure 3f). Figure 3g shows selective regional programmability of fluorescence within the wrinkled surface induced by UV radiation, in agreement with the emission color change shown in the CIE chromaticity diagram. The LSCM and optical images show that such surfaces possess unordered topologies and rough appearance prior to and post UV treatment. Intriguingly, in the irradiated area, TPEOH became an inactive chromophore, and the violet flower could be identified under a UV lamp by the naked eye. Furthermore, light‐driven tests against the functionalized bilayer demonstrated that the synergistic control over the wrinkle shape and fluorescence can be realized by switching on/off the NIR source due to the previously confirmed mechanism. The conversion, that is irreversible photocyclization and reversible fluorescence, of the stimuli‐responsive bilayer surface was obtained by applying either 365 nm UV or 808 nm NIR irradiation, resulting in a tunable multicolor wrinkled dual‐pattern.

The misuse of the developed technology to forge anti‐fake labels or unlawfully deceive verification tools frequently occurs for daily trade banknotes, luxury articles, identity cards, and pharmaceuticals; this damages significant social ecological chains and poses great threats to mankind throughout the world. Although a considerable range of security tags has been reported, there are still various challenges in providing appreciable technologies due to the deterministic processes, encoding capacity, and so on.^[^ ^18^, ^23^ ^]^ As previously described, the presented pattern of photodynamic fluorescent wrinkles has many advantages: 1) facile yet robust processes; 2) development of biomimetic fingerprint‐like structures via unpredictable micro‐topologies; 3) observation of the programmable fluorescence drawing upon UV excitation, which allows ready authentication of labels with naked eyes; and 4) temporal alteration of the security information of fluorescence and wrinkling coding by NIR irradiation (**Figure** **4**a). Such dynamically tunable memory surfaces exhibit an intriguing promise in breaking the limitations caused by static or single models.

**Figure 4:**
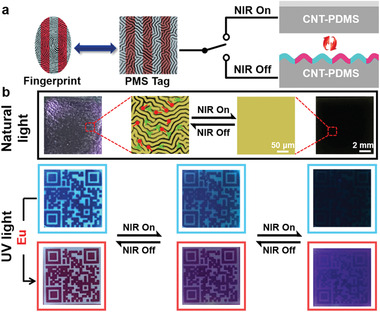
Pattern memory surface serving as a full‐color security tag. a) Demonstration of switchable fluorescence and wrinkle information within the memory surface exhibiting colored fingerprint‐like structures. b) Images of the QR codes with or without the Eu^III^ complex under natural and UV light regulated by an NIR source (1.5 W cm^−2^). Note: The LSCM image indicates that the marked minutiae, including ridge bifurcation (green color) and ridge ending (red color), of the wrinkles can be highly altered by switching on/off the NIR source. Scale bars: 50 µm and 2 mm.

Random and unpredictable wrinkled layers were obtained by subjecting the supramolecular network to a simple thermal treatment at 100 °C for 3 min and subsequently cooling it to room temperature. After irradiating this network by 365 nm UV light through a QR code‐shaped photomask, significantly fluorescent wrinkles were obtained, which could be readily decoded through direct scanning (Figure 4b). Furthermore, the exposed location can be tuned to emit aubergine fluorescence by adding the Eu^III^ complex to the supramolecular solution prior to spin coating; this enables alternative morphological encryption for the generation of security labels. Note that the incorporation of regional fluorescence into wrinkles plays an important role in boosting the anti‐counterfeiting standards. However, aside from the two‐channel fluorescence emissions, the information within the surfaces was further enriched because of the generated wrinkles. As demonstrated in the LSCM image, the fluorescent wrinkles contain minutiae including ridge bifurcation and ridge ending, which is similar to the biological fingerprint feature. Upon noninvasive NIR irradiation, the minutiae of the wrinkled patterns disappeared and the emission of the patterned fluorescence declined. Upon extended exposure to NIR irradiation, the emission information of the fluorescent QR could not be obtained and the fluorescent QR was difficult to recognize under a UV lamp owing to the increased molecular motions and thermal expansion. The memory tags returned to their initial morphologies after turning off the NIR source; this revealed the outstanding on‐demand exhibition of the proposed dual‐pattern.

In summary, we demonstrated a facile strategy using an AIE‐exhibiting supramolecular network as the skin layer for constructing a photodynamic memory surface with a regulated fluorescent and wrinkle pattern. TPEOH was used in the fabrication of the wrinkled pattern and led to acid‐sensitivity and controlled fluorescence of the bilayer system, which possesses a distinct luminous mechanism and provides a hydroxyl group that forms a hydrogen bond with the tertiary amine group of poly(St‐*co*‐Dm) to form a dynamic supramolecular network. In situ selective UV photodirect writing offers ordered fluorescence to the unpredictable wrinkling surfaces and thus realizes programmable patterning. Based on the photo‐to‐thermal effectivity, the noninvasive NIR light not only darkens the cyan fluorescent pattern, but can also erase wrinkles in the bilayer system. Multiple message encryption/decryption cycles based on the external NIR stimulus exhibited fascinating flexibility of this dual‐pattern. The proposed strategy without complicated methodology is fundamentally important to implement security information, providing an important platform to engineer anti‐counterfeiting tags, and expected to inspire more studies related to AIE‐mediated wrinkling surfaces with photodynamic tunable memory patterns in the near future.

## Conflict of Interest

The authors declare no conflict of interest.

## Supporting information

Supporting Information

Supplemental Movie 1

Supplemental Movie 2
