# Supplementary material for: Photodynamic Pattern Memory Surfaces with Responsive Wrinkled and Fluorescent Patterns
Source: Adv Sci (Weinh). 2020 Oct 14;7(22):2002372. doi: 10.1002/advs.202002372 (PMC7675060; doi:10.1002/advs.202002372)
Supplement: Supplementary file 1 — Supporting Information [file ADVS-7-2002372-s001.pdf]

WILEY-VCH

© 2020 Wiley-VCH GmbH

ADVANCED SCIENCE
Open Access

Supporting Information

for *Adv. Sci.*, DOI: 10.1002/advs.202002372

Photodynamic Pattern Memory Surfaces with Responsive Wrinkled and Fluorescent Patterns

*Shuai Chen, Tianjiao Ma, Jing Bai, Xiaodong Ma, Jie Yin, Xuesong Jiang**

1. Materials and Characterizations

1.1 Materials: Europium(III) chloride hexahydrate and 4,4'-dihydroxybenzophenone were purchased from Adamas-Beta Co. Ltd. (Shanghai, China). Styrene, n-butyl acrylate and 2,2-azobisisobutyronitrile (AIBN) were purchased from Sigma-Aldrich (Shanghai, China). Carbon nanotube (CNT) and 2-(dimethylamino)ethyl methacrylate (Dm) were provided by Macklin Chemical (Shanghai, China). And all other chemicals were obtained from Hitachi Chemical (Shanghai, China).

1.2 Characterizations: The ^{1}H nuclear magnetic resonance (^{1}H NMR) spectra were recorded with Bruker Avance III HD spectrometer (500 MHz, Bruker, Germany), 298K. Average molecular weights of copolymers were determined by means of gel permeation chromatography (GPC, LC-20A, Shimadzu, Japan), using tetrahydrofuran as an eluent at a flow rate of 1.0 mL min^{-1} with a combination of two columns (Shodex, KF-802 and 804, 300 × 8 mm) and equipped with a RID-10A differential refractive index detector. The atomic force microscopy (AFM) images were observed by utilizing a scanning probe microscope (Nanoscope III, Digital Instruments), which operated in tapping mode with silicon cantilevers (with a force constant of 40 Nm^{-1}). All the fluorescence spectra were recorded using a steady-state & time-resolved fluorescence spectrofluorometer (QM/TM/IM, USA PTI Industry) equipped with a temperature control system. And glass transition temperature (T_g) was measured by modulated differential scanning calorimety (DSC, TA-Q2000, USA) at a scanning rate of 5 ^{o}C/min. Super resolution multiphoton confocal microscopy (STED) was conducted on Keyence VK-X150. Wrinkling patterning surfaces were recorded by profile measurement microscope (VF-7510, KEYNCE, Japan) and laser scanning confocal microscopy (LSCM, LEXT VK-X1000, Keyence, Japan).

2. Experimental Section

2.1 Synthesis of Tetrakis(4-(hydroxy)phenyl)ethylene (TPEOH) and Tetraphenylethene (TPE)

The synthetic procedure of the aggregation induced emissive (AIE) molecule TPEOH is presented in **Scheme S1**. Briefly, zinc dust (3.92 g, 60 mmol) was suspended in a 250 mL three-necked flask with dry THF (60 mL). Then, $TiCl_4$ (3.32 ml, 30 mmol) was dropwise added to the above zinc suspension within ice-salt baths, followed by room temperature stirring for 30 min and refluxing for 2 h, cool-down to 0 °C and being supplemented with anhydrous pyridine (1.2 mL). Subsequently, 4,4'-dihydroxybenzophenone (4.28 g, 20 mmol) in anhydrous THF (40 mL) was added to the aforementioned solution. After reflux reaction overnight, the yielding crude product was purified according to the following procedures: washing with 10% K_2CO_3 solution, extraction with DCM and dehydration with Na_2SO_4. After evaporation of solvent, the product was purified by silica gel column chromatography (ethyl acetate: petroleum ether = 1:3) to give TPEOH as the purple solid product powder (3.5 g, 89%).

Following the similar procedure described for TPEOH afforded a pure solid product in a yield of 93%. The chemical structure was verified by ^{1}H NMR (**Figure S1**).

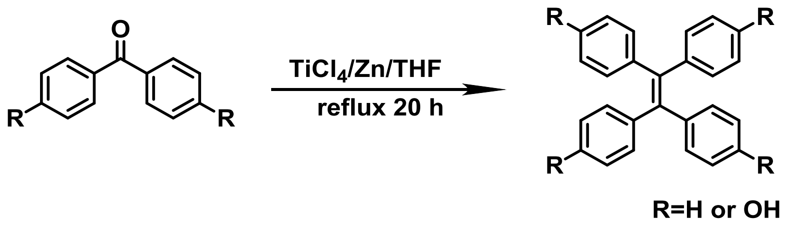

Scheme S1. Synthetic scheme for preparation of AIE molecules.

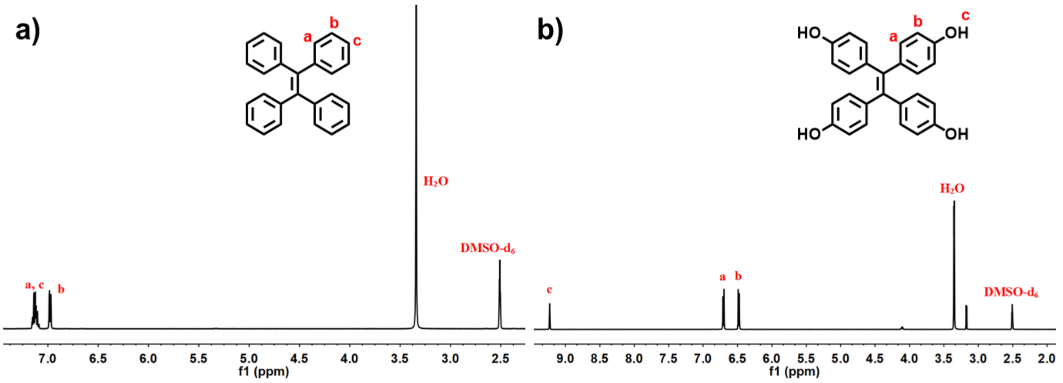

Figure S1. ¹H-NMR spectra of the synthesized a) TPE and b) TPEOH in DMSO-d_6 solution.

2.2 Synthesis of Copolymers.

The synthetic procedure of copolymer was depicted in **Scheme S2**. In detail, n-butyl acrylate (1.28 g, 1 mmol), styrene (2.08 g, 2 mmol) and AIBN (1 wt.% total monomer weight) were successively added into 15 mL of dry 1,4-dioxane under the protection of nitrogen atomosphere and stirred at 70 ^{o}C for 12 h. After cooling to room temperature, the mixture was precipitated in cold n-hexane. The filtered powder was dried in vacuum condition at 50 ^{o}C for 24 h to result in copolymer poly(Ba-*co*-St) as white solid product (yields: 89 %). The respective constituent ratio of the copolymer is confirmed by integrals in ^{1}H NMR spectra.

Dm-loaded copolymer was synthesized similarly. Herein, Dm (4.5 g, 3 mmol), styrene (6 g, 5.8 mmol) and AIBN (105 mg, 1 wt.% total monomer weight) were dissolved in 15 mL anhydrous 1,4-dioxane and stirred at 70 ^{o}C for 12 h under N_2 atmosphere. The following preparation procedures of Dm-containing copolymer (poly(St-*co*-Dm) were the same as above.

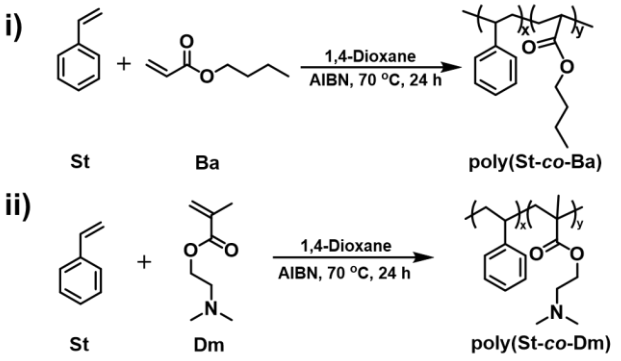

Scheme 2. The synthesis route to copolymer i) poly(St-*co*-Ba) and ii) poly(St-*co*-Dm).

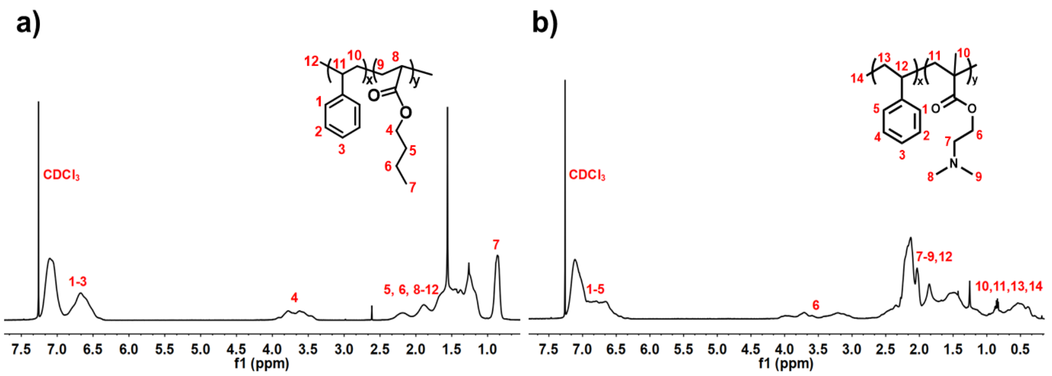

Figure S2. ^{1}H NMR spectra of a) poly(St-*co*-Ba) and b) poly(St-*co*-Dm) in $CDCl_3$ solution at 298 k.

Table S1. Summary results of the utilized copolymers in this work.

Sample	Mole ratio		GPC Data		
	Feed	Actual	Mn(kDa)	Mw(kDa)	PDI
**Poly(St-*co*-Ba)**	2:1	2.1:1	32.6	51.0	1.57
**Poly(St-*co*-Dm)**	2:1	1.6:1	16.7	24.0	1.43

2.3 Synthesis of the functional Europium complex

General procedure: According to **scheme S3**, about 1.0 mmol of $EuCl_3$ dissolved in 10 mL of methanol was added to a solution of thenoyltrifluoroacetone (4.0 mmol, TTA) in methanol (20 mL) while stirring. The clear solution became turbid after its pH value was tuned to 9.0 by adding $NH_3{\cdot}H_2O$. This mixture solution was stirred at reflux for 4 h, during which time the white precipitates generated. Finally, the crude product was purified by filtration, washed with cold petrol ether, and dried in vacuum to obtain the title complex as a white solid (yield: 83%).

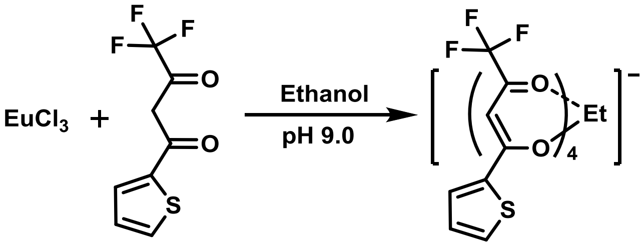

Scheme S3. Schematic diagram of the preparation of the EuTTA complex.

2.4 Preparation of Polydimethylsiloxane (PDMS) Substrate and CNT-containing PDMS

Silicone elastomer (Sylgard 184, Dow Corning) was used to fabricate elastomeric PDMS sheets. Detailedly, the base and crosslinking agent were thoroughly mixed in petri dished at a determined weight ratio (15:1), followed by being degassed at room temperature for 2 h. Subsequently, the mixture was heated to 70 ^{o}C for 4 h to result in a cross-linked PDMS elastomer substrate and then divided into square pieces with 1 cm length of side.

Moreover, CNT was deftly introduced into base agent to give NIR-responsive PDMS sheets. In detail, single-walled CNTs at varying weight (1.5 mg, 7.5 mg and 15 mg), 15 g of PDMS and 10 mL of normal hexane were added into a beaker. After sonication for 24 h, the well-dispersed mixture was dried in a vacuum oven at 70 ^{o}C overnight to remove the residual solvent. The obtained CNT-doped PDMS base was moved to a petri dish and then mixed with 1 g of curing agent. Finally, the CNT-PDMS substrates were obtained by solidifying the degassed solution at 70 ^{o}C for another 4 h.

2.5 Fabrication of dynamic wrinkled pattern

To obtain AIEgens-mediated dynamic wrinkle pattern, 5.12 mg of TPEOH was firstly dissolved in 1 g of anhydrous THF. Then 80 mg of poly(St-*co*-Dm) dissolved in toluene (1 g) was added to the mixture. After directly complexing for 5 min, the filtered solution of poly(St-*co*-Dm)@TPEOH was spin-coated onto the CNT-PDMS sheet to give in a bilayer system. The samples were subjected to thermal treatment for 3 min and cooled down to room temperature to result in multifunctional wrinkling pattern with fluorescence.

2.6 Preparation of tunable fluorescent wrinkle pattern

The toluene/THF mixed solution of poly(St-*co*-Dm)@TPEOH or poly(St-*co*-Dm)@TPEOH@Eu was spin-coated on the PDMS sheet. Upon heating for 3 min and cooling down, wrinkled surfaces with disorder topological structures occurs. To obtain patterned fluorescence wrinkle, the samples were then irradiated by 365 nm UV (15 mW/cm^2) at 70 ^{o}C for 30 s through figurate photomasks, respectively.

Statistical Analysis. Significant differences in fluorescent ratio of the top layer obtained by super-resolution multiphoton confocal microscopy (STED) between any two groups were evaluated using Student's *t* test.

3. Results and Discussion

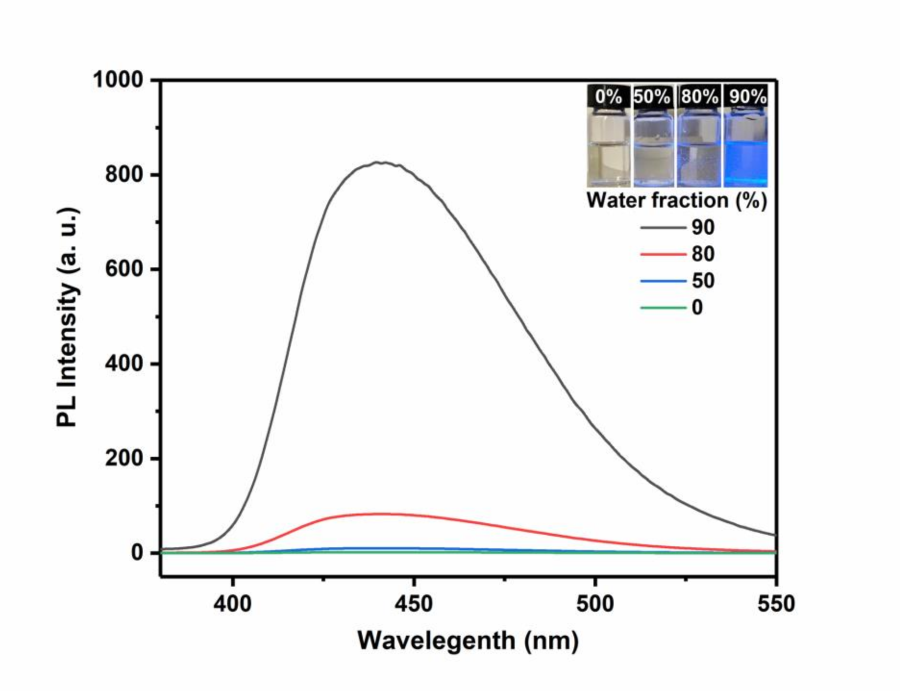

Figure S3. Fluorescence emission spectra of TPEOH in THF/water mixed solvents containing different water fractions. Excitation: 330 nm.

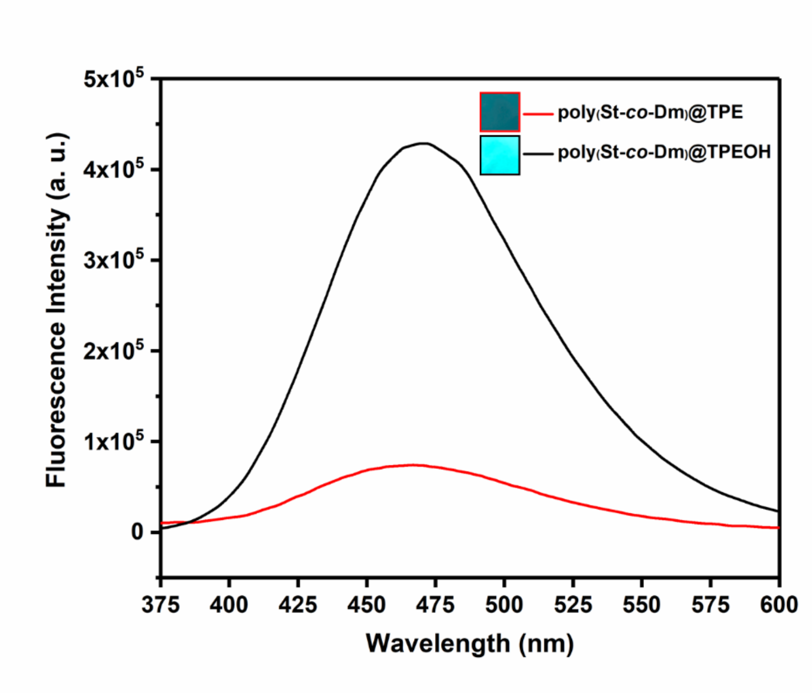

Figure S4. Fluorescence emission spectra of 1 wt.% AIE-loaded poly(St-*co*-Dm) films (Excitation: 330 nm) and their corresponding photographs under a UV lamp (365 nm).

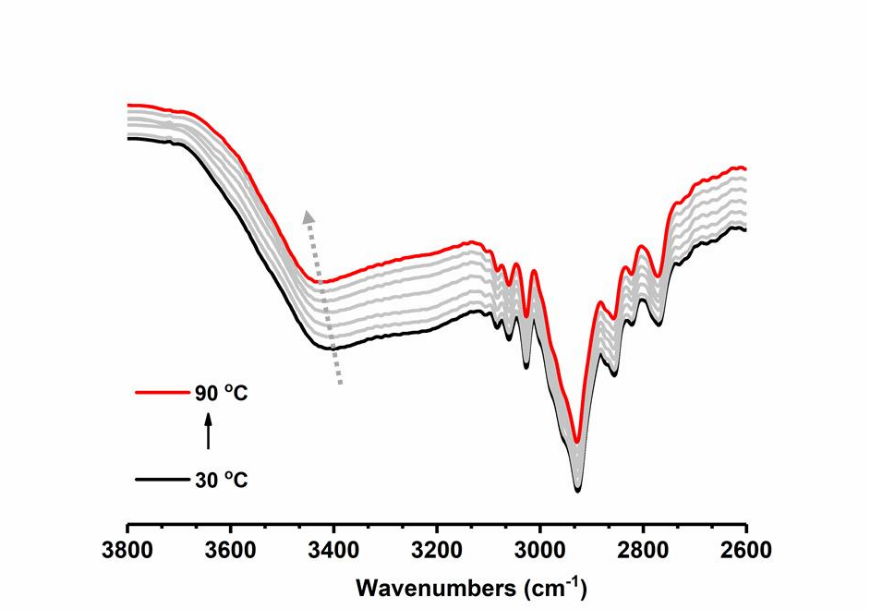

Figure S5. Temperature dependent FT-IR spectra of poly(St-*co*-Dm)@TPEOH. The experimental temperature was 30, 40, 50, 60, 70, 80 and 90 ^{o}C, respectively.

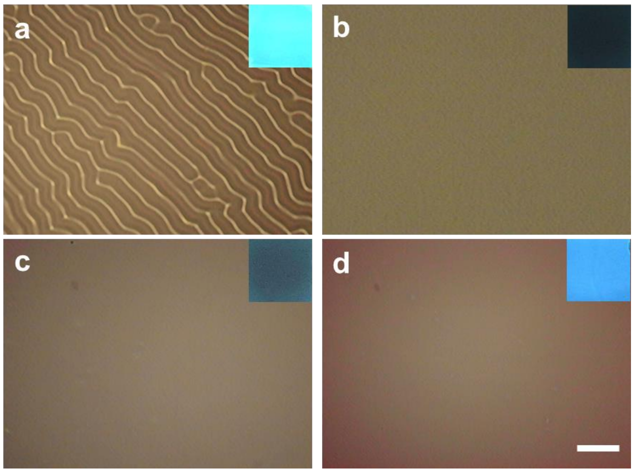

Figure S6. Optical image of a) poly(St-*co*-Dm)@TPEOH, b) poly(St-*co*-Dm), c) poly(St-*co*-Dm)@TPE, and d) poly(St-*co*-Ba)@TPEOH post thermal treatment (70 ^{o}C for 3 min). The insets are photographs of supramolecular films under 365 nm UV light irradiation. Scale bar: 50 μm.

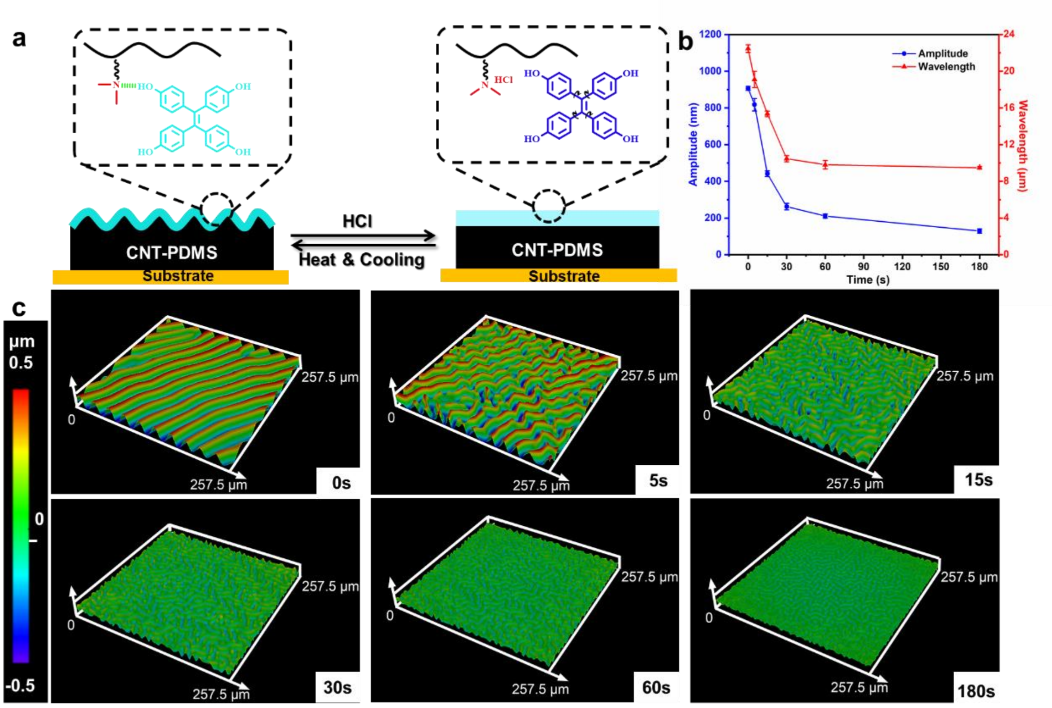

Figure S7. Dynamic characteristic of the wrinkles responsive to hydrogen chloride. a) Schematic illustration of erasure/regeneration of wrinkling surfaces during acid treatment. b) Amplitude (A, blue line) and wavelength (λ, red line) of wrinkles as a function of HCl vapor for a series of time. c) LSCM images of erasure process of the wrinkled structure after HCl treatment for different times.

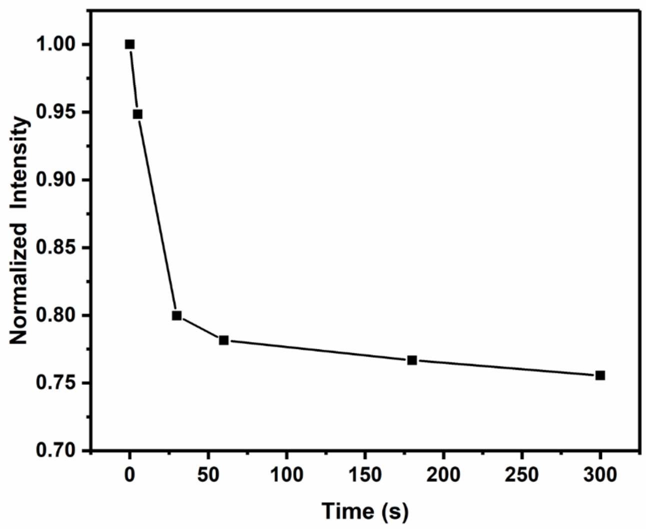

Figure S8. The fluorescence intensity plot of TPEOH-doped copolymer film at 460 nm in the atmosphere containing HCl for different times (Excitation: 330 nm).

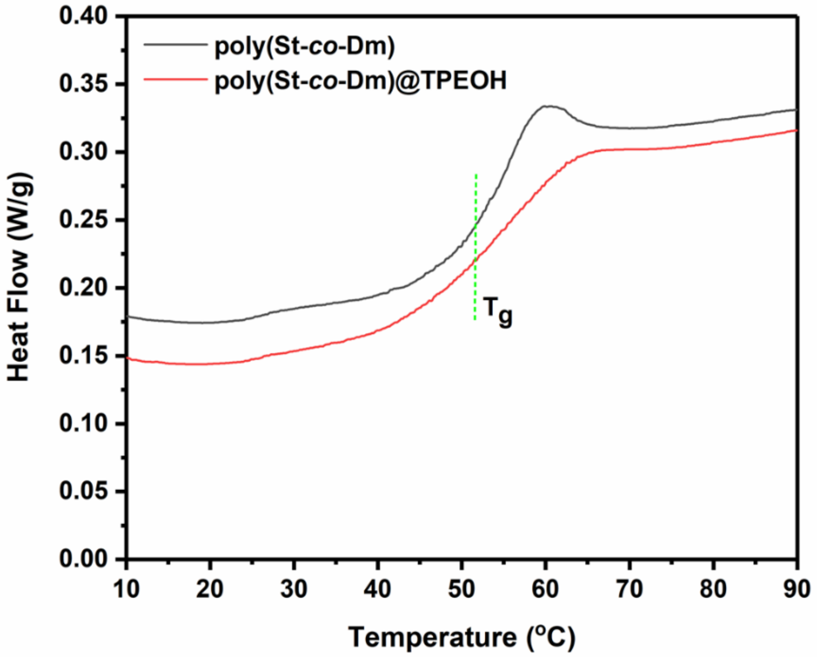

Figure S9. Differential scanning calorimetry (DSC) curves of poly(St-*co*-Dm) and poly(St-*co*-Dm)@TPEOH (containing 1.0 wt.% TPEOH) at heating rate of 5 °C/min.

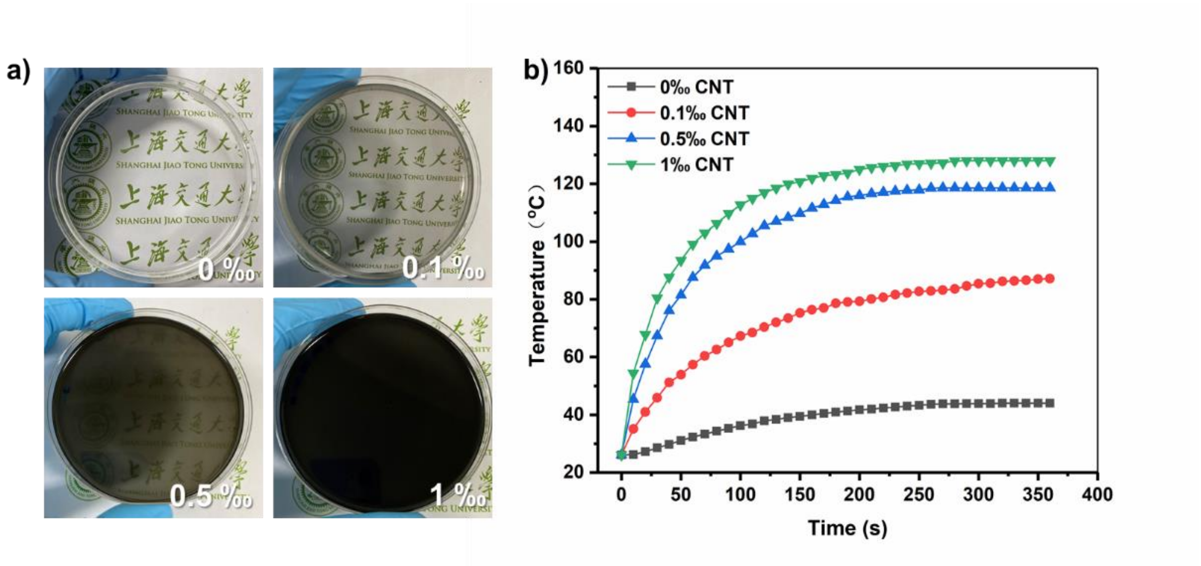

Figure S10. a) Images of CNT-PDMS containing various concentrations of single-walled carbon nanotubes. b) Temperature variation curves of CNT-PDMS surfaces during NIR irradiation (808 nm, 1.5 W/cm^2).

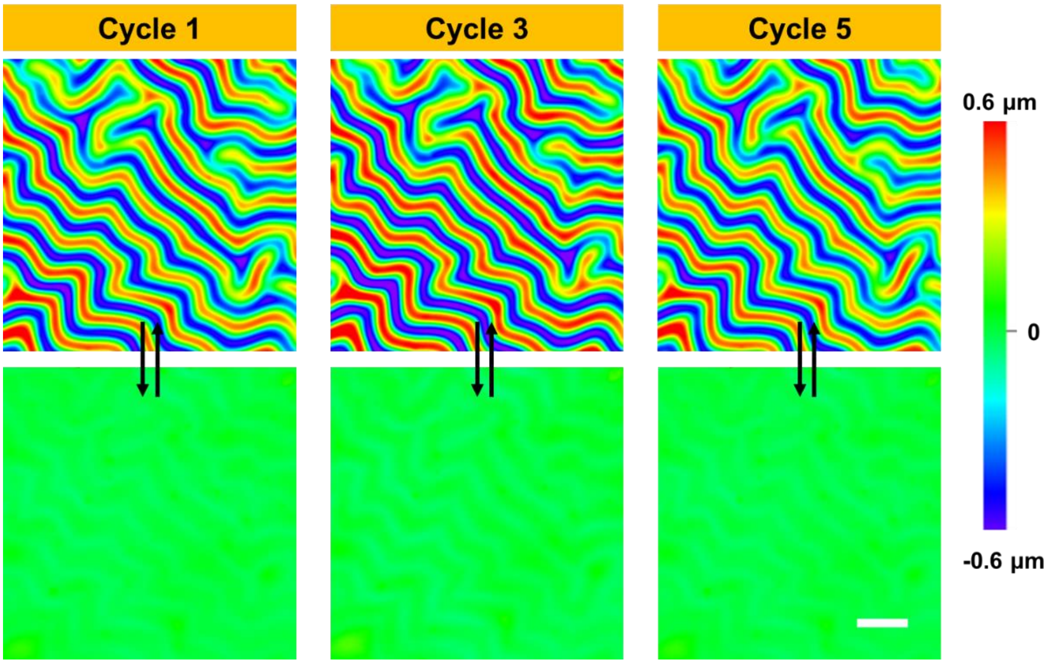

Figure S11. LSCM images of extinction/formation cycles during the on/off irradiation by infrared light. The NIR light intensity is 1.5 W/cm^2. Scale bar: 30 μm.

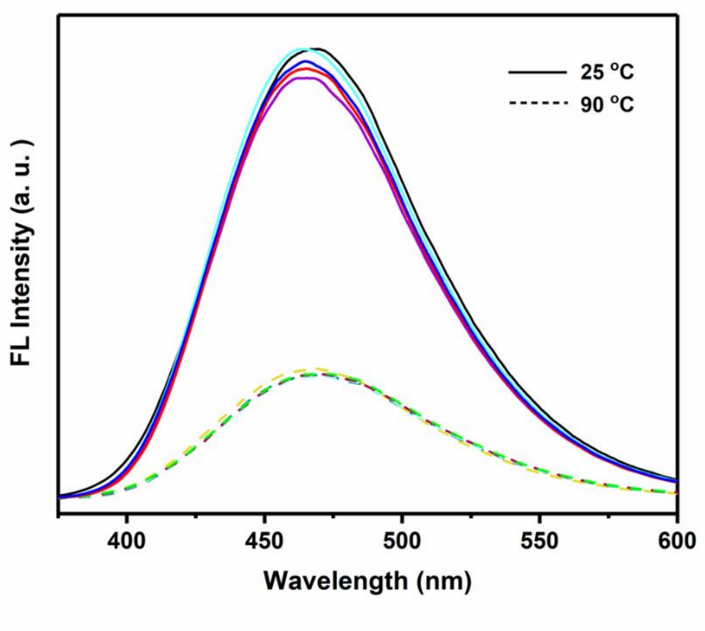

Figure S12. Fluorescence spectra of 1 wt.% TPE-doped copolymer film at 25 °C and 90 °C under the protection of nitrogen by switching several cycles (Excitation: 330 nm). The NIR light intensity is 1.5 W/cm^2.

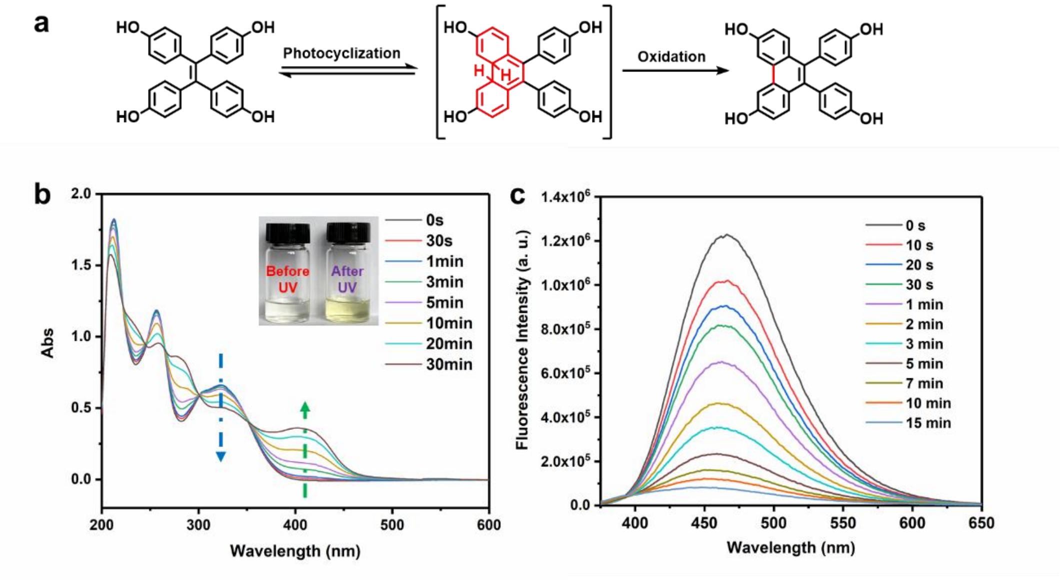

Figure S13. a) Schematic illustration for photo-oxidation process of TPEOH. b) UV spectra of TPEOH in CH_3OH solution and c) fluorescence spectra of 1 wt.% TPEOH-doped poly(St-*co*-Dm) film for different irradiation times of 365 nm UV light (Excitation: 330 nm).

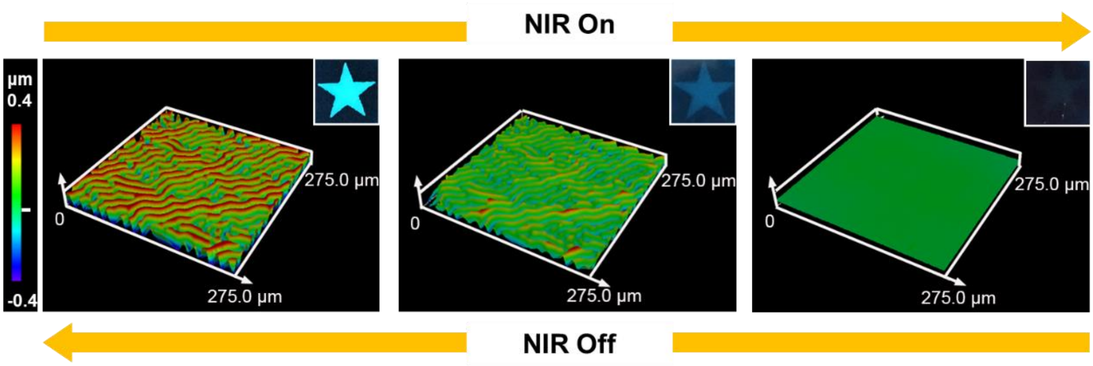

Figure S14. Reversible changes of star fluorescent wrinkle dual-patterning by switching the on/off irradiation of NIR.

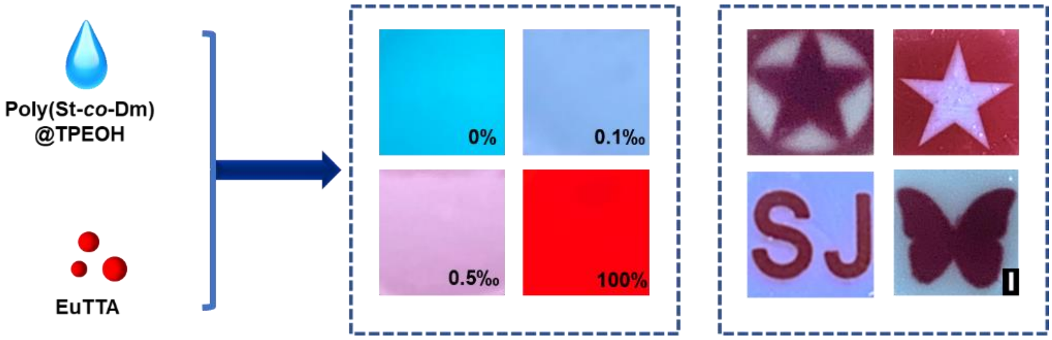

Figure S15. Photographs of poly(St-*co*-Dm)@TPE films doped with a series of rising contents of EuTTA coordination compound and 0.1 wt.‰ EuTTA-loaded supramolecular network post UV light (365 nm, 15 mW/cm^2) direct writing through mask under a UV lamp. Scale bar: 2 mm.

Movies

Movie S1. Video of the reversible disappearance/formation behavior of the random wrinkles during the on/off irradiation by infrared light.

Movie S2. Video of the fluorescent pattern's disappearance/formation behavior under NIR irradiation.
